# Abstracts and Gleanings

**Published:** 1881-12-20

**Authors:** 


### ABSTRACTS AND GLEANINGS.

**Belladonna as an Antidote to Opium Poisoning.**—Dr. J. P. McGee, of Trenton, Tennessee, reports to the West Tennessee Medical Society, that on the 18th of March, George B——, a robust man about 35 years of age, took for suicide 30 grains of opium (which I learned from the druggist was a moist, good article), and drank about 12 ounces of whisky, as he said, to "make the 'pizin' work better."

The fact was not known for about an hour, at which time I was called. I found him sitting on the side of the bed, a good deal annoyed at being interrupted, protesting against having a doctor and declaring his determination not to submit to any treatment. Persuasion and threats were alike unavailing. He refused absolutely to take or submit to anything. So, as I was unable to procure the assistance necessary to coercion, I could but stand by and watch the "pizin work." Two hours after the inhibition of the drug, he said to me, "Well, Dr., you can 'let in your blade,' now. I think I've got enough to beat you, anyhow. So, to satisfy you, you may try your hand."

With drowsy nonchalance he swallowed for me a considerable quantity of tepid water and ipecac, and at different doses near ʒij of sulphate of zinc (!) with no more signs of emesis than if I poured it in the slop bucket.

Finally (in two to two and a half hours from the time of taking the drug) in spite of cold douching, walking or rather dragging, whipping with wet towels, etc., he lapsed into profound narcotism. By this time four other physicians, all my seniors, were there. We held a "council of war." I proposed atropia hypodermically. No one present, except Dr. Caldwell, had seen it used, and all but him objected that it would but increase the narcotism.

Nevertheless I determined to use it. So I promptly injected in the arm $\frac{1}{8}$ gr. atropia sulph., an amount which they all assured me would kill him if the opium didn't. Very soon, however, the pulse, before almost imperceptible, grew stronger, the respiration less labored and more frequent. Presently, the pupils—before almost occluded, so great was the contraction—began slowly to dilate; later, the death-like paleness of the face began to show some color, so that in ten or fifteen minutes he would flinch or move away when pinched or pricked with a pin, and in about twenty-five or thirty minutes vomited freely, and could be sufficiently aroused to give intelligent answers.

In about one-half to three-quarters of an hour more, however, narcotism came on again and was soon as profound as before. Another $\frac{1}{8}$ gr. of atropia was injected, with results as before, but more prompt and more continuous, showing in a more marked degree the characteristic effects of atropia.

This time narcosis returned no more, and in three days he was discharged.

The pupils remained unduly dilated for more than twenty-four hours. The dryness of the mouth and throat was very distressing.

I gave this unusually large dose of atropia upon the ground that the dose of antidote should be somewhat proportionate to the amount of poison taken. He had taken at once fifteen full doses of opium. I gave five full doses of atropia at each injection, the two being equal in proportion to two-thirds the amount of opium taken.

I am satisfied now, although the reasoning was good, that the quantity was much larger than necessary.—*Miss. Val. Medical Monthly.*

**Prolapse of the Ovaries.**—The monthly recurrence of the periodical flow fills the ovaries with catamenial blood, and they become heavy from their own weight, and sink low down in the pelvis, from this cause. They oftentimes can be felt by introducing the index finger into Douglass' cul de sac. If this congested state remains they do not return to their place in the abdomen, but from disease remain in the unnatural position, and morbid phenomena ensues, generally some uterine lesion acting either as a cause, or is the result of this prolapse. Laceration or the retroflexion of the womb, or subinvolution, and sterility are frequent csuses, as well as sexual excess, and ovarism, the left ovary is usually dislocated, when only one is out of place. In the symptoms, our first attention is called to pain in loco-motion, which at each movement, catches the prolapsed ovary between the sacrum and the uterus and giving rise to intense suffering. It is a pain similar to that often experienced when riding horseback, if mashing the testicle on the pummel of the saddle, nauseating and very sickening, and pale faced, with cool shivering.

The genito-crural nerve which runs down the thigh, is often the tract of pain in this trouble. A loaded rectum adds to the pain, as the hard fæces act roughly on the sensitive parts. Pain confined to one groin, and in coition, are other symptoms of this malady. All this is attained by great hebetude, and extremely low spirits. Menorrhagia, dysmenorrhœa, and uterine trouble in general, most always forces these patients to seek aid.

Dr. Goodell prescribes first in these troubles: Brom. pot. grs. xxx, and tinct. digitalis gtts. x ter die, in ℥ ss infusion gentian comp. He does this with the view of keeping down any erethism of the sexual organs, as both medicines are anaphrodisiacs. In two weeks then, he uses alteratives, and chloride of ammonium and bichloride of mercury are his favorites here; he also lauds chloride of gold and sodium. Blisters often repeated over each ovary, scarification, carbolized iodine, suppositories of iodoform and belladonna, and last, but by no means the least, *hot water.* Hard pessaries and those which are too short to stretch out Douglass' cul de sac do more harm than good; the air rubber pessaries are best suited to this condition of prolapsed ovary with some uterine displacements. Goodell uses an elastic ring pessary. Dr. H. E. Campbell, of Georgia, recommends the placing of the woman in the knee-breast posture, almost three times a day, with knees ten inches apart, and thighs perpendicular to the bed. If the woman in this position opens the vulva with one hand, the air rushes in, and after the method of Sims. lifts the vaginal walls and sags the abdomen and contents down, but the woman cannot very well spare one hand and Dr. Campbell recommends the use of a glass tube open at both ends and to come out at the vulva.

Abdominal braces are used for supporting the external parietes, and Dr. Goodell explains their action thus: "By pressing the abdominal wall upward and inward the brace forms a shelf on which the vicera rest, and thus take off a portion of the load from the womb and its ovaries. By virtually narrowing the pelvic aperture, it lessens the space into which the viscera tends to crowd, and to that extent, protects the pelvic organs. By bringing the pelvis backward, it makes the axis of the upper strait lie more obliquely to the axis of the trunk; and the sum of the visceral pressure now converges, not in the pelvic basin, but on the portion of the abdominal wall, lying between the symphysis pubis and the umbilicus."

Dr. Weir Mitchell has combined with the knee-breast posture, rest in bed, massage, electricity, and forced feeding, and the daily forcing up of the ovaries, with the atmospheric method above described. The ovaries under this treatment, after fat and health are restored, generally stay up. If, after all, the ovaries remain prolapsed and give trouble, the dernier resort, is extirpation. This does not unsex a woman, nor make her less a wife or a mother, while it takes away all hopes of future offspring. (Goodell.)—DR. AUSTIN, in North Carolina Medical Society.—[N. C. Med. Journal.

**A Successful Operation of Gastrotomy for Intussusception.**—Harry B. Estill, M. D., Tazewell C. H., Va., says: On the 3d day of October, 1881, Oscar Holly—a strong, robust, muscular man—was suddenly seized with a severe abdominal pain, of a griping or spasmodic character, referred to the neighborhood of the umbilicus. My father, Dr. J. M. Estill, was immediately summoned, who exhausted all the known remedies for mechanieal obstruction of the bowels, viz: catharsis, copious enemata, opium, etc., without result. He continued to treat the patient upon general principles, until Saturday morning—five days after his attack began—when, according to previous arrangements, he started for the meeting of the Medical Association of Virginia at Old Point Comfort, and I, who had been absent in the meantime, saw him, at two o'clock on Saturday morning, for the first time. I found him with coldness of the skin, prostration, distressed countenance, persistent constipation, constant vomiting entirely of a stercoraceous character, abdomen tender and very tympanitic, with a slight tumor, dull upon percussion, immediately to the left of the umbilicus. From these symptoms, and the history of the case, I diagnosed intussusception or invagination, and decided that an operation was the only remedy by which to give my patient a chance for life.

Accordingly, on Monday morning, the 10th day of October, and the eighth day of Holly's illness, I, with the kindly assistance of my friends Drs. A. S. Hoffard and Thomas Witten, performed the operation of gastrotomy, opening the abdomen between the umbilicus and pubes, along the linea alba, five inches, through the peritoneum, and passing my fingers to the supposed point of obstruction, I found the diagnosis to be correct, the invagination occurring in the small intestines (jejunal) immediately to the left of the umbilicus. The gut *below* the obstruction was very much engorged, and presented every appearance of acute enteritis. The adhesions were pretty firm, but with moderate force they were separated. The wound was closed with sutures, the peritoneum being included; but, on the fourth day, as considerable peritonitis had supervened, the sutures were removed and plaster substituted therefor. The peritonitis very readily yieldied to treatment; the temperature did not exceed 100° F., and the pulse did not reach 100. I should have mentioned, that the anticeptic method (Lister's) was used throughout.

On this, the 22d day after the operation, the patient is able to walk, and in a few days will be able to engage in his usual avocation.—[Va. Med. Monthly.

**The Best Position for Women in Labor.**—Dr. G. J. Engleman (*Trans. Amer. Gynæ. Society*, 1880, p. 266) after a very careful study of this subjeet, reaches the following conclusions: (1) In the ordinary labor case, the patient should be given greater liberty and should be permitted to follow the dictates of her natural instinct in regard to her movements more freely than is now customary. (2) In the earlier stages of labor the parturient must be guided in her actions, and in the position assumed, by her own comfort and dictates of her own instinct. Not only is this the invariable rule among the savages, but it was also warmly advocated by the shrewd and observing obstetricians of the past, and by those eminently practical and successful midwives of old. (3) The ease with which the parturient women of uncivilized people avoid the dorsal decubitus, the modern obstetric position, at the termination of labor, is sufficient evidence that it is a most undesirable position for ordinary cases of confinement; and I am convinced that the thinking obstetricians will soon confirm the statement not unfrequently made by the ignorant but observing savage, by Negro and Indian, that the recumbent position retards labor and is inimical to easy, safe and rapid delivery. (4) In the ordinary labor cases the expulsion of the child should be expected in an inclined position, kneeling, squatting, or semi-recumbent, in bed or lap, as is done by the great majority of uncivilized people for the following reasons: (*a*) These positions permit the free use of the abdominal muscles. (*b*) The force of gravity does not counteract the expulsive effort, as in the recumbent position, nor does it unite with it too freely and hasten labor unduly, as in the erect posture. (*c*) With the assistance of a rope, stake or other support the parturient can vary the inclination of the body and correct the labor, hasten or retard the descent of the child and relieve the pain, changing the axes of the body and throwing the fœtal head towards the sacrum or symphysis. (*d*) Injury to the soft parts is less liable to occur in these positions, if we may accept the rapid getting up and freedom of our Indian squaw from all uterine diseases as proof of this statement. (5) Of these positions the semi-recumbent is the most serviceable, and should be adopted as the obstetric position in all ordinary labor cases. It is preferable to the kneeling or squatting: (*a*) As being more convenient and comfortable, not exposing the person and not being objectionable to the modesty of the patient. (*b*) As affording more rest and not being tiresome, which is a serious objection to the kneeling and squatting positions as applicable to the tender female of our civilization. (*c*) The semi-recumbent position in bed, the body at an angle of forty-five degrees, the hips resting on a hard mattress, thighs well flexed, is the easiest, most comfortable, and appears to afford the greatest relief and the greatest freedom from pain, coupled with the greatest effect of the uterine contractions, relaxations of all the parts and free play of the abdominal muscles. (*d*) The pelvis is more readily fixed in this position. (*e*) The perineum has a certain support which does away with the questionable proceeding of supporting the perineum during the expulsion of the head and shoulders, by which more harm than good is usually done.—*Detroit Lancet.*

**Sodium Phosphate in Habitual Colic.**—Dr. R. N. Taylor, in the Medical Herald, writes:

"It has fallen to my lot to have been beset with a number of patients who would persist in having colic at the most inopportune moments imaginable; first one, and then the other, then still another one, these colicky friends of mine were constantly interfering with my professional engagements, and what was more important stlll, with my hours of rest. Without entering into a discussion of the pathology of these cases, I beg leave to present in brief the history of one of the worst I have ever seen, together with the treatment, which resulted in completely warding off the attacks in this case, as in all others in which it has been carried out:

"Mrs. H—, aged seventy-two years, for fifteen years has been the subject of attacks of cramp-colic, recurring at first every two or three months, but for six years past coming on every two weeks, sometimes every week; has taken nearly everything in the materia medica, both at the hands of regulars and quacks, without any benefit, the attacks continually recurring, and that without any regard to errors of diet, coming on at nearly regular intervals, no matter how careful or how particular she might be in regard to her diet.

"During the paroxysms of colic the pain is most atrocious, accompanied by cramping pain in the extremities, nausea, vomiting, etc., to such an extent as to demand my immediate presence, armed with the hypodermic syringe.

"The patient was put upon the use of phosphate of soda, grs. xxx., ter in die, before meals and—the fees stopped. She had no more attacks of colic. If she omits the use of soda for a length of time, say six or eight weeks, she will have a few premonitory twinges, the precursors of a more severe attack; but immediately obtaining a supply of the drug, she is safe so long as she continues to use it, and for some time afterwards.

"This is not an isolated case, but one of several that could be adduced in favor of the use of phosphate of soda in the colic habit; but it is deemed useless to multiply instances upon so trivial a matter. Suffice it to say that I have never failed to see the administration of phosphate of soda followed by a complete cessation of the attacks of colic, and my experience in the use of this drug has now become quite extensive.

"In these cases I generally begin with thirty grains three times a day, and if that amount produces much irritation of the bowels, indicated by frequent small discharges, attended perhaps by some tenesmus, I diminish the dose to twenty or fifteen grains. It is to be administered before meals, from a half to one hour, in a glass of water, and when thus dissolved it is not at all unpleasant to the taste."—*Drug. Circular.*

**The Limit of Danger in the use of Anæsthetics.**—In this connection certain investigations recently made by Professor Paul Bert, and reported in the Gazette des Hopitaux, are interesting and instructive. He made a great number of experiments on dogs, mice and sparrows, with the following anæsthetics: ether, chloroform, amylene, bromide of ethyl, and bichloride of methylene. His principal object was to determine the differences which separate anæsthetic doses from fatal doses; that is, the proportions in which the anæsthetic agent becomes mingled with the blood in order that it may induce only anæsthesia, or cause the death of the animal. The number of grammes of the anæsthetic to 100 liters of air necessary to produce these effects is as follows:

| | DOG. | | MOUSE. | | SPARROW. | |
|---|---|---|---|---|---|---|
| | Anæsthesis. | Death. | Anæsthesis. | Death. | Anæsthesis. | Death. |
| Ether | 37 | 74 | 12 | 25 | 18 | 40 |
| Chloroform | 15 | 30 | 6 | 12 | 9 | 18 |
| Amylene | 30 | 55 | 15 | 30 | 30 | 60 |
| Bromide of ethyl | 22 | 45 | 7 5 | 15 | 15 | 30 |
| Bichloride of methylene | 21 | 42 | 12 | 20 | 12 | 24 |

It is seen from these figures that the quantities which cause death are exactly double those that are necessary for the production of anæsthesia. Now, in practice, when chloroform is, for example, poured on a napkin, it constantly happens that a patient is made to inhale twice as much of the agent as is necessary to induce anæsthesia; in other words, the risk is run of getting what *might* prove a fatal dose. It is certainly an alarming consideration that, to produce death, only double the dose necessary for anæsthesia is required. All who have occasion to administer the valuable but dangerous agents for anæsthetical purposes should bear this fact constantly in mind.—*Eclectic Med. Jour.*

**Small-Pox in Richmond.**—There are, at this writing, not more than 72 cases—if, indeed, so many—of small-pox and varioloid in

Richmoud—including the cases in the Small-pox Hospital, as well as those in private quarters From first to last, of the present epidemic, the disease has been almost confined to the negroes and lower white population. But now, even these elements of the community are having their eyes open to the necessity of vaccination, and are either submitting themselves to the advice of their family physicians, or else are crowding the offices of the public vaccinators. There need be no longer any apprehension as to the further material spread of the disease.

If there were doubts in the minds of any in this community heretofore as to the protective value of vaccination, such doubts can no longer have foundation. But another equally valuable lesson learned by some is, that vaccination is not a protection for a life period in many cases. Revaccinations, at short periods—even every three or four years—is a prudential measure to be adopted in well regulated families. Some of the patients who have had severe cases of varioloid, during the present epidemic, claim to have been, and give evidences of having been, successfully vaccinated only five or six years ago. Repeated vaccinations every four or five years are not apt to do harm; failure to be as frequently revaccinated may result in great injury to a community as well as to the negligent individual.

This seems to have been an epidemic year for small-pox in many sections of the United States. Let those communities which have not been afflicted take warning, and *at once* submit themselves to vaccination and revaccination. Such a course, if adopted at once, will render it wholly unnecessary to think of quarantines.—*Virginia Medical Monthly.*

**Restoring the Heart's Action When it has Ceased to Beat.**—I do not remember what induced me to kill a mouse by a blow upon the head, and rip it open to see the heart beat. It did not. I pricked it with a needle and set it a-going. It stopped after a time. Then I gave it a second prick, and a few pulsations were distinctly seen. When I was in petticoats my father was sent for to see a girl in a fit. He was out, and when he came home he was informed of the fact. "How long ago, and any second message?" Being told, he thought he need not go. My mother suggested he "ought to go," which he did. He found the girl dressed in her grave-clothes and "laid out" upon a linen-covered table. He examined her and found some warmth over the heart. He ordered hot water to be brought (not scalding hot), and poured it into a jug; tore her shroud open, stood on a chair, and poured a continuous stream of hot water, until the throbings of the heart were distinctly seen. That girl was the mother of several children before I left Scotland, in 1848. My mother used to laugh, and take her share of the credit of her restoration to life.

An old man here, Robert Robinson, several years before his death, took a fit, and apparently expired upon the floor, where he was lying, pulseless and breathless. The heart had ceased to beat, and I was told that "he was beyond any doctor's power now." I felt some warmth over the heart, and tried my father's remedy; and to the wonder of spectators, the septuagenarian revived and lived several years afterward. Hot water can easily be obtained, and no one can object to such an experiment,—[J. C. Reid, M.D., British Medical Journal—*Southern Practitioner.*

**Ipecacuanha in Jaundice.**—Professor Rossbach had observed a certain catarrhal condition in the trachea in the cat, in which it was found to be exceedingly constant; and if he injected pecacuanha into the veins of the animal, it became at once very much increased, and very much less tenacious. That was exactly what was wanted here—something which would enter the blood and act upon the mucous in the bile-ducts, and thus allow the bile to push its way into the duodenum. It had been asked what doses of ipecacuanha were used. He had himself had no experience of the use of ipecacuanha in jaundice. In fact, it was only a month or two since he learned of it, and he had had no cases d[illegible]tly under his treatment since.

Dr. Ewart had mentioned that a quarter of a grain to a grain was used in India. It depended upon the nausea.

Lately, also, Dr. Hook, of Bombay, recommended it in very large doses in the same way as for dysentery. He gave a sixth of a grain of morphia beforehand, and then thirty grains of ipecacuanha half an hour afterwards as a bolus; and he had found cases of jaundice improve very satisfactorily in a very short time, and one case in twenty-four hours that had resisted other treatment. The other plan of treatment, as Dr. Ewart had mentioned, was that of continued small doses. Then, in regard to the action of euonymin, he had not tried it in jaundice, but in other cases of intermittent liver disorder in consequence of malaria in men who had been out in India, say three grains of euonymin, made up into a pill, every second or third night, followed by a little Carlsbad water in the morning. Usually he told his patients to take a large draught of the water in the morning after the pill, and on the other mornings the same quantity of the water taken in small sips, as they did at Carlsbad, so that a tumblerful should last them till they had finished dressing, the water to be previously heated to the warmth of warm tea, so that they could comfortably sip it. This combination of euonymin with Carlsbad water gave very good resnlts indeed in these cases of biliary disorder depending upon chronic malarious poison.—[Med. Times.

**Pneumonia Aborted with Quinine.**—Dr. Munson, in Virginia Medical Monthly, reports a number of cases aborted or cut short with quinine. He says:

"Give quinine boldly, and continue it at intervals of four or five hours, until the pulse approaches the normal standard. I would advise that the first dose should not be less than 20 grains for an adult, if decided febrile action is present, to be repeated in doses of six to ten grains every four or five hours, until 40 grains are taken, if necessary, to reduce the pulse. I would not advise more than 40 grains to be given in the first 24 hours. Less (say 30 grs.) will ordinarily suffice.

The medicine must be persisted in from day to day until the symptoms yield. I have been compelled to continue it in some cases for four or five days, but this has been very rarely necessary. Usually it will only be required in diminished doses after the first day of treatment—the rule being simply to increase the dose according to the violence of the febrile reaction. It is my custom to give the first dose during the latter hours of night, or very early in the morning, and to precede it by three or four hours with a full dose of calomel. The sulphate restrains the mercurial from exciting hypercatharsis; the calomel resists the sedative influence of the quinine, besides possessing other virtues unnecessary to repeat.

Give the quinine early the first night the patient is seen. Do not wait until the lungs are blocked up with blood—and above all, do not postpone it until that final, fatal delirium appears. The patient is then usually doomed. Do not, however, despair even then—for this treatment will sometimes rescue him when hope has almost fled.

When the pulse is below the normal standard, do not give large doses of quinine. The dose ought not to transcend three or four grains in four or five hours, nor should more than 12 or 15 grains be given in 24 hours, watching closely its effects. In adynamia, small doses might depress too much.

Weigh your medicine carefully. I attach a doubtful value to the experience of any one who pursues the uncertain and reprehensible custom, too prevalent among physicians, of *guessing* at the doses of their medicines. The pauper, convict and jail-bird have all of their medicines weighed or measured. Surely the worthy and respectable recipients of our skill deserve no less care.

MODE OF ADMINISTERING QUININE.—I prefer to give it simply diffused in cool water. If this is impracticable, after being ground finely, it may be made into pills with syrup or gum arabic (U. S. Ph.), or placed in the capsule of lichen before mentioned, or mixed in coffee. I know no moderate quantity of this latter vehicle will interfere with the salutary action of the remedy, as Briquet (Op. cit. p. 306) erroneously supposes.

Be sure that your quinine is genuine. I use Powers' and Weightman's exclusively. The foreign articles in my hands have often failed.—*Ex.*

**Catching Cold.**—Few persons take cold who are not self-consciously careful or fearful of the consequences of exposure. If the attention is wholly diverted from self as in efforts to save life at a fire, or in the water, the effects of chill are rarely felt. This seems to indicate that the influence exerted by cold falls upon the nervous system. If the immediate effects of the contraction of the surface vessels by cold and the coincident dilation of the internal vessels sufficed to produce an inflammation, then surely we would have such inflammations all of the time. But as a fact, when the vascular system is healthy and that part of the nervous apparatus which controls the calibre of the vessels acts properly, then any disturbance of the equilibrium of the vessels, which may have been produced by cold, will be speedily readjusted. This being granted, everything depends upon the nervous system. Now consciousness is one element in the production of cold. and when this is wanting the phenomena is not likely to occur. Hence it is that persons who do not cultivate the fear of cold-catching are, as a rule, not subject to this infliction. This also explains why the habit of wrapping up tends to keep up a morbid susceptibility. The mind, by this fear-begetting precaution, keeps the nervous system on the alert for impressions of cold and the centres are, so to speak, panic stricken even when a slight sensation appears. Many of the sensations of heat or cold which are experienced by hyper-sensitive persons have not external cause. They are ideal in origin and ideal in fact."—*Detroit Lancet.*

**New Treatment for Syphilis.**—The Paris correspondent of the London Lancet writes: M. Martineau has published the result of a large number of cases of syphilis treated by a new method at the Hopital Lourcine. The preparation employed consists of a mixture of powdered peptone, chloride of ammonia, and bichloride of mercury, which are dissolved in water and glycerine. In order to have a standard solution which shall contain five centigrams (.02 grain) in a gram, the following proportions are taken:

℞ Powdered peptone (Catillion)............ grs. ix,
Chloride of ammonium,.................. grs. ix,
Bichloride of mercury,.................. grs. vj, M.

These are dissolved in glycerine, seventy-two grams; water, twenty-four grams. This solution, which the author calls "normal," further diluted with five parts of distilled water, is of such strength that an ordinary French hypodermic syringeful represents ten milligrams, or one-fifth of a grain of corrosive sublimate. The solution is injected subcutaneously, and the dose employed by M. Martineau has varied from two milligrams (1-25 grain) to ten (1-5 grain) of bichloride of mercury. Altogether one hundred and seventy-two patients have been under observation, and a total number of three thousand eight hundred and thirty-eight hypodermic injections made. No abscesses or sloughs have ever followed the operation; sometimes a defective injection has given rise to a lump, but this has always rapidly disappeared. There is never either stomatitis or salivation, even with one-fifth of a grain of the mercuric salt daily.—*Med. and Surg. Rep.*

**Treatment of Chronic Ulcers of the Leg.**——Courty's procedure in leg-ulcers is as follows: First, the sore is cleansed by washing with antiseptic solutions, as carbolic acid, salicylate of sodium, thymol, chloral, permanganate of potassium. Then, later, soap or an alkali is used to remove the debris and excess of epidermis. Proceeding then to the stimulation of the granulations, Coufty spreads red precipitate ointment (strength of 1-50 to 30) on fenestrated pieces of linen, and then lays these over the ulcer. Over this cotton compresses are laid in sufficient thickness to absorb all the purulent discharge. Over this is placed a slightly compressive rubber bandage. Courty says that patients soon learn to put this bondage on for themselves twice daily, which is advisahle. He thinks that the red precipitate ointment, combined with the warmth and moisture, lead to active hyperæmia of the granulations, with absorption of the callous edges of the ulcer. When the granulations begin to grow excessive, and the border of the ulcer has softened down, it is time to begin the attempt at cicatrization. Courty thinks that care should be taken at this stage to avoid too rapid drying of the newly-formed epidermic layers, and the consequent puckering or fissuring about the border. Moist warmth, he thinks, best effects this.

Among the means used to favor cicatrization, Courty recommends an aromatic wine, containing, occasionally, some salt of copper or arsenic. Transplantation, also, according to Courty, is advantageous when there is an extensive granulating surface. When the aromatic wine cannot be employed, nitrate of silver may be used, or, if this is too strong, Sydenham's laudanum. Frequently the application of the aromatic wine is followed by dressing with an ointment containing one part tinct. opii to ten parts simple cerate. This opium ointment is almost invariably employed together with compresses to finish up the cicatrization.—Med. Times.

**Treatment of Hernias of Long Standing.**—The following conclusions are reached by M. Thiry, in a paper on the above subject presented by him to the Royal Belgian Academy of Medicine: (1.) Old hernias of large extent, constituting a variety of eventration, are susceptible of reduction in most cases. (2.) The large volume of hernia is never a contradiction to its reduction, although it necessitates the adoption of certain precautions, and the employment of considerable time. (3.) The diminution in the capacity of the abdominal cavity, in old hernias, is never antagonistic to a slow, methodical, and progressive taxis. (4.) By slowly re-entering the abdominal cavity, the extended parts gradually resume their former place. (5.) The best method of reduction is the "compressing taxis," which consists in only restoring organs to their natural position after they have been relieved, by pressure, of any vascular engorgement. Those organs which effected an exit last should be first replaced. (6.) In this variety of hernia, an elastic truss, adapted to the gradually decreasing size of the tumor, should be attached to an abdominal belt. (7.) When the hernia has been completely reduced, the projecting knob of the truss, with properly shaped convexity, should penetrate the ring and adapt itself to its dimensions.—Bulletin de L'Academic Royale de Medicine de Belgique.—[Buffalo Med. Journal.

**Coca a Cure for Morphinism.**—La Independencia Medico quotes the following case: A lady had been in the habit of alleviating her sufferings with morphine, of which drug she finally took sixteen grains a day. Thirty hours after having taken her last dose she was found in great anguish, excitation and inquietude. During the night chloral hydrate and iodide of potassium were given to allay the excitation and produce sleep. The next day she was very weak and restless, hardly able to speak, and tormented with vomiting; the pulse was 150. The fluid extract of coca was administered in doses of a tablespoonful. The first dose had but little effect. The second was followed by a wonderful change; the pulse fell to 85, the countenance assumed color and animation, and the vomiting ceased. The patient began to speak, and was in excellent spirits. She slept almost half of the following night, awoke refreshed, with a pulse of 75, took breakfast, and digested it well. She continued to improve, rode in a carriage for quite a distance, and left the city next day, taking with her an eight-ounce bottle of coca, which remedy she continued to take in diminishing doses. When she ceased taking it she was enjoying good health, without the use of morphine.—*N. Y. Med. Record.*

**Alcohol.**—The power of alcohol, says Bartholow, to coagulate albumen, to suspend the activity of the unorganized ferments, and to destroy minute organisms, lies at the foundation of its external uses. It is a most efficient hemostatic to restrain bleeding from wounded surfaces. "As an antiseptic dressing to wounds, to prevent the entrance of the germs of putrefaction, to check suppuration, and to promote healing, it has scarcely been inferior to the much-vaunted carbolic acid. It is an efficient means for procuring local refrigeration of an inflamed joint or swelling. Injected under the skin in the neighborhood of painful nerves, it has no inconsiderable anodyne power. This property may be utilized for the relief of myalgia and lumbago. It is more efficient than water, used by the method now known as aquapuncture. Enlarged tonsils, hypertrophied thyroid, and glandular swellings may often be slowly reduced and made to disappear by the parenchymentous injection of alcohol. This method is also applicable to the treatment of uterine fibroids."—[Med. Record.

**Dietetic Treatment of Intestinal Fluxes.**—Under the above heading Dr. E. N. Chapman, of Brooklyn, gives a very interesting and rational article in the September number of the Virginia Medical Monthly. In all cases under this head he prescribes milk, with an addition of one-sixth part lime water for the principal diet. In many cases he finds it necessary to begin with a small dose of calomel, followed by castor oil, and sometimes he combines tonics, etc., according to indications; but he finds this particular diet useful in all cases, and his reports of cases show rapid cures effected in many obstinate chronic cases, as well as in the more impressible acute cases. The diarrhœa of typhoid fever is no exception to the rule, but he always prescribes the diet above mentioned in this disease, because of its simplicity and rationale. The treatment recommends itself as worthy of trial.—*Detroit Lancet.*

**Capsulotomy.**—The great question in extracting cataracts has always been as to the best method of dealing with the *capsulæ lentis.* Grœfe taught the absurd plan of attempting to make a triangular section with two cystotomes curved so as to adapt them best to enter, one at each angle of the corneal section; and, commencing at the inferior margin in the vertical meridian, cut upwards and outward toward the opposite angle to that at which the cystotome was introduced, thus incurring the risk of producing an ectropia which would greatly endanger the safety of the eye. The peripheral laceration, which was introduced by the writer in 1876, and since practiced successfully by Knapp and others, allows so much cortical matter to remain as to make it desirable to practice central incision with the point of the knife in making the corneal section.—*Med. Herald.*

**Mr. Jefferson Davis,** in his work, "The Rise and Fall of the Confederacy," refers to Documents put forth by the Secretary of War and Surgeon General, U. S. A., and shows, by them, that there were, in round numbers, 270,000 Union prisoners, of whom 22,000 died; while there were 220,000 Confederate prisoners, of whom 26,000 died; that is to say, the mortality among the Union prisoners was less than 9 per cent.; while that among the Confederate prisoners was more than 12 per cent. These figures are official. The mortality of the prisoners was thus 25 per cent. greater in the prisons of the United States.—*American Med. Bi-Weekly.*

**New Test for Albumen in the Urine.**—Dr. Henry Luffmann exhibited a new method of testing for albumen by the use of glacial phosphoric acid in fine powder, a few grains of which are to be added to the fluid to be tested.

Dr. Neff inquired whether the reaction of the urine would have any effect upon the test.

Dr. Leffmann said that he did not think it would, because the test is used in sufficient quantity to overcome any alkalinity, and, as it diffuses very slowly, it would show the albumen at once.—*Medical Times.*

**Comparative Proportion of Physicians to Inhabitants in Different Countries.**—The latest calculations give the following proportion of physicians to each ten thousand inhabitants in various countries:

| | |
|---|---|
| France, | 2.91, |
| Germany, | 3.21, |
| England, | 6.06, |
| Austria, | 6.10, |
| Italy, | 6.10, |
| Switzerland, | 7.06, |
| United States, | 16.24. |

—*Medical Times.*

**Hot Rectal Douche.**—Dr. James R. Chadwick, in Gynecological Society Medical Times, writes upon "Hot Rectal Douche," and gives a number of interesting cases of uterine and associated rectal disease relieved by this method, and urges its use as a means of relief in backache, painful defecation, rectal pain, burning in abdomen and pelvic effusion.—*Med. Times.*

